# Association Between Oral Frailty and Health Indicators Using Data from the 2023 Korea National Health and Nutrition Examination Survey: A Cross-Sectional Study

**DOI:** 10.3390/healthcare14142113

**Published:** 2026-07-14

**Authors:** Seol-Hee Kim

**Affiliations:** Department of Dental Hygiene, Konyang University, Health Sciences Building, 158 Gwanjeodong-ro, Seo-gu, Daejeon 35365, Republic of Korea; ableksh@konyang.ac.kr; Tel.: +82-42-600-8449

**Keywords:** age factors, KNHANES, mental health, oral frailty, quality of life, sarcopenia

## Abstract

**Highlights:**

**What are the main findings?**

- Among Korean adults, oral frailty was found to be associated with aging, socioeconomic vulnerability, poorer physical and mental health, and reduced quality of life across the life course.
- Oral frailty was observed not only in older adults but also across earlier stages of adulthood.

**What are the implications of the main findings?**

- These findings underscore the importance of recognizing oral frailty as a multidimensional health condition that develops in adulthood, rather than as a consequence of old age.
- The findings highlight the need for preventive oral health strategies and community-based oral health programs from early and middle adulthood to identify individuals at risk before substantial functional decline occurs.

**Abstract:**

**Background/Objectives:** In this study, we aimed to investigate age-specific prevalence and patterns of oral frailty among Korean adults and examine its multidimensional associations with physical and mental health and health-related quality of life, from a life-course perspective. **Methods**: This cross-sectional study included 4459 adults aged ≥25 years from the 2023 Korea National Health and Nutrition Examination Survey. Oral frailty was defined as the presence of ≥4 deficits across 9 clinical and behavioral indicators. Associations of oral frailty with chronic diseases, appendicular skeletal muscle mass (ASM), psychological symptoms, and health-related quality of life were examined across age-stratified cohorts (25–44, 45–59, 60–74, and ≥75 years). **Results**: The prevalence of oral frailty was significantly higher in older age groups (8.2% at 25–44 years to 48.5% at ≥75 years, *p* < 0.001). Frail participants showed significantly higher prevalence of hypertension and diabetes and lower ASM, compared with non-frail participants. Difficulty pronouncing words and toothache experience showed the strongest contributions to the overall oral frailty score (*p* < 0.001). Oral frailty was also significantly associated with higher perceived stress and generalized anxiety, particularly among younger adults aged 25–44 years. Health-related quality of life was consistently lower in frail participants than in non-frail participants across all age groups (*p* < 0.001). **Conclusions**: Oral frailty is a multidimensional indicator associated with systemic aging, metabolic health, psychological distress, and reduced quality of life across the life course. These findings support the need for integrated, life-course-oriented oral health interventions beginning in early adulthood to prevent functional decline and promote healthy aging.

## 1. Introduction

The health profile of adults has become increasingly complex due to rapid global aging, encompassing chronic diseases, nutritional deficiencies, musculoskeletal weakening, and cognitive decline [1,2]. Among these, oral health is a pivotal component of systemic frailty; however, it is frequently overlooked in early adulthood health interventions. Recent research has recognized oral frailty as a multidimensional, potentially reversible stage of functional decline that reflects the interplay between physiological aging and oral health behaviors [3,4].

Extensive literature establishes that tooth loss and impairments in mastication or swallowing are not merely localized dental issues but are significantly associated with poor nutritional status and physical decline [5,6]. Oral diseases have been reported to be associated with systemic frailty, which in turn has been associated with cardiovascular disease and metabolic disorders [7,8]. Furthermore, the link between oral function and neurological health is gaining increasing attention; fewer natural teeth and impaired chewing ability have been associated with cognitive impairment, and oral functional problems are significantly linked to psychological stress, anxiety, and depression [9,10]. Studies using standardized instruments, including the Generalized Anxiety Disorder-7 (GAD-7), have reported significant associations between anxiety symptoms and self-perceived oral health [11]. In this context, oral frailty has emerged as a holistic health marker reflecting declining oral function, physical frailty, functional disability, and diminished quality of life [12]. It arises from complex interactions between physiological factors and behavioral indicators, including fewer natural teeth, denture use, oral pain, and infrequent dental check-ups [13,14]. Oral frailty is being increasingly recognized as an important indicator of healthy aging because of its close association with systemic functional decline and social participation [15,16]. Countries such as Japan have incorporated oral frailty management into integrated healthcare policies to promote healthy aging [17,18]. Korean studies have predominantly focused on older adults often emphasizing late-stage manifestations of oral frailty, such as denture use, rather than earlier functional decline [19,20]. Despite emerging studies using national data, there remains a critical lack of evidence regarding the associations of oral frailty across different adult age groups, limiting the evidence available to inform age-specific oral health strategies.

Furthermore, previous studies have primarily operationalized oral frailty using functional indicators, such as chewing ability, swallowing function, tongue pressure, oral diadochokinesis, and tooth loss [15,16,17,18]. In comparison, the broader associations of oral frailty with psychological and social health outcomes across the adult life course have been less extensively investigated. This knowledge gap impedes the development of proactive, rather than reactive, oral health policies that could mitigate health risks before they lead to permanent disability.

Therefore, in this study, we aimed to investigate age-specific patterns of oral frailty and their multidimensional associations with physical, mental, and socioeconomic factors among Korean adults aged ≥25 years using data from the 2023 Korea National Health and Nutrition Examination Survey (KNHANES). Additionally, we aimed to examine the relative contributions of individual oral health components within the composite Oral Frailty Index (OFI) across the adult life course. By extending the scope of analysis beyond older adults and the traditional focus on functional oral health indicators alone, we sought to provide evidence for the development of life-course-oriented oral health interventions and multidisciplinary strategies for the early identification and management of oral frailty.

## 2. Materials and Methods

### 2.1. Data Source and Study Population

This study involved a cross-sectional secondary analysis of raw data from the 2023 KNHANES [13,14]. KNHANES is a nationally representative surveillance system set up by the Korea Disease Control and Prevention Agency (KDCA) under the National Health Promotion Act to monitor the health and nutritional status of the Korean population. The survey consists of a health interview, a health examination, a nutrition survey, and an oral examination, providing comprehensive and nationally representative data for health-related research. The KNHANES employs standardized data collection protocols to ensure the reliability and validity of the survey data. Health interviews are conducted by trained interviewers using standardized questionnaires, and health and oral examinations are performed by qualified healthcare professionals according to standardized operating procedures in mobile examination centers. Before participating in the survey, all personnel receive standardized education and field training. Quality-control procedures, including utilization of standardized manuals, periodic retraining of survey personnel, equipment calibration, field supervision, and periodic data verification, are implemented throughout the survey by the KDCA.

**Figure 1:**
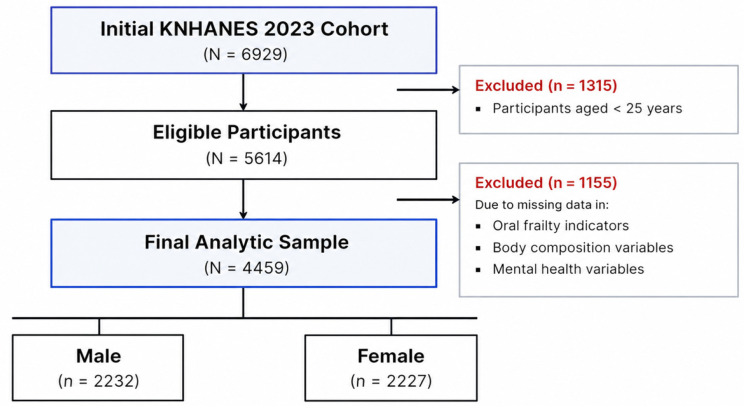
Flowchart of participant selection.

A multistage stratified cluster sampling design was employed to ensure the generalizability of the findings to the non-institutionalized South Korean population. Of the initial 6929 participants, 1315 individuals aged <25 years were excluded. Of the remaining 5614 participants, 1155 with missing data on oral frailty indicators, body composition, or mental health variables were excluded, resulting in a final analytic sample of 4459 participants (2232 male and 2227 female), as illustrated in Figure 1. All participants provided written informed consent before participation. The KNHANES protocol was approved by the KDCA Institutional Review Board (approval No. 2022-11-16-R-A). This study was reviewed and ethical approval was waived by the Institutional Review Board of Konyang University (IRB No. KYU-2025-08-019).

### 2.2. Measures

#### 2.2.1. OFI

Oral frailty was operationalized as a cumulative deficit index using nine KNHANES indicators adapted from established models [5,15,17,21]. Components included: (1) poor self-rated oral health, (2) denture use, (3) chewing difficulty, (4) oral pain, (5) no dental check-up within the past year, (6) <20 natural teeth, (7) masticatory discomfort, (8) speaking difficulty, and (9) toothbrushing <2 times/day. Each item was dichotomized (1 = deficit, 0 = no deficit). Based on previous studies on oral frailty, participants with an OFI score of ≥4 were classified as having oral frailty for the descriptive analyses [5,19,21].

This cutoff was adopted from the Oral Frailty Index-8 (OFI-8) and was selected based on previous international and Korean oral frailty literature, thereby facilitating comparison with previous studies [5,17,19,21].

#### 2.2.2. Physical and Mental Health Variables

Physical health variables assessed included self-rated health, activity limitations, and diagnosed comorbidities (hypertension, diabetes, dyslipidemia). Body composition—specifically total body water, fat-free mass, and appendicular skeletal muscle mass (ASM)—was measured via multi-frequency bioelectrical impedance analysis (InBody 970; Biospace, Seoul, Republic of Korea) following standard Asian protocols [22]. Mental health was assessed through evaluations of perceived stress and depressive symptoms and by using the Health-related Quality of Life Instrument with 8 Items (HINT-8), which covers eight dimensions (climbing stairs, pain, vitality, working, happiness, sleeping, depression, and memory). Generalized anxiety was assessed using the validated Korean version of the GAD-7, which consists of seven items assessing nervousness, uncontrollable worrying, excessive worrying, difficulty relaxing, restlessness, irritability, and fear of impending harm [23].

### 2.3. Statistical Analysis

Statistical analyses, including descriptive analyses, group comparisons, correlation analyses, and multivariable linear regression analyses, were performed using the Complex Samples module of IBM SPSS Statistics version 28.0 (IBM Corp., Armonk, NY, USA), incorporating sampling weights, primary sampling units, and stratification variables to account for the complex survey design and ensure national representativeness. The prevalence of oral frailty across age groups (25–44, 45–59, 60–74, and ≥75 years) was compared using Rao–Scott chi-square tests. Continuous variables were analyzed using the complex samples general linear model with Bonferroni or Scheffé post-hoc tests. Spearman’s rank correlation analysis was performed as an exploratory analysis to examine the associations between oral frailty-related factors and health indicators. Multivariable linear regression analysis was performed to assess the relative contribution of individual OFI components to the overall oral frailty score, adjusting for demographic and health-related covariates. Because the OFI is a composite index derived from predefined oral health components, the regression model was used to evaluate the relative contribution of each component to the composite index, rather than to identify independent predictors of oral frailty. Prior to the regression analysis, multicollinearity was assessed using variance inflation factors and tolerance values, and the independence of residuals was evaluated using the Durbin–Watson statistic. Significance was set at *p* < 0.05 (two-tailed). Potential sources of bias were minimized through the nationally representative multistage sampling design of the KNHANES, standardized data collection procedures, and adjustment for potential confounding variables in the multivariable analyses.

## 3. Results

### 3.1. General Characteristics and Oral Health Status of the Study Population

Figure 1 presents the participant selection process. Of the 6929 participants initially enrolled in the 2023 KNHANES, 4459 participants were included in the final analyses after applying the eligibility criteria.

Oral health and functional indicators showed significant age-related deterioration (*p* < 0.001; Table 1). Subjective poor oral health and chewing difficulty were higher in the ≥75-year age group (44.6% and 34.8%) than in the 25–44-year age group (22.5% and 7.4%). Clinical measures followed a similar pattern, with fewer natural teeth (15.93 ± 9.46) and more frequent denture use (37.7%) observed in the ≥75-year age group than in the 25–44-year age group (27.90 ± 2.11 and 0.1%, respectively) (*p* < 0.001). Functional impairment, including chewing and speaking difficulties, significantly worsened with age (all *p* < 0.001). Notably, toothache experience (30.7%) and oral examination rates (44.6%) peaked in the 45–59-year age group, whereas the ≥75-year age group reported the lowest examination participation (24.9%). Toothbrushing frequency significantly varied across age groups, with the highest frequency observed in the 60–74-year age group (2.30 ± 0.93) (*p* < 0.001; Table 1).

The prevalence of oral frailty differed significantly across age groups, ranging from 8.2% in the 25–44-year age group to 48.5% in the ≥75-year age group (*p* < 0.05; Table 1). Across most age categories, male participants exhibited a higher prevalence than female participants did (*p* < 0.05), except in the ≥75-year age group, where the difference was not significant (*p* = 0.589). Socioeconomic disparities were evident; a lower education level (middle school or less) was consistently associated with a higher prevalence of oral frailty across all age groups (*p* < 0.05). Similarly, Medical Aid recipients showed a markedly higher prevalence, compared with National Health Insurance (NHI) subscribers, in the 25–44-, 45–59-, and 60–74-year age groups (*p* < 0.05). Notably, these socioeconomic and sex-based associations weakened in the oldest age group (≥75 years), where insurance type (*p* = 0.118) no longer showed significant differentiation (Table 1).

### 3.2. Physical Health Indicators and Oral Frailty

Oral frailty was significantly associated with compromised physical health across all age groups (Table 2). Frail participants reported higher rates of activity limitations (9.9–26.9%) and subjective poor health (25.8–33.3%), compared with non-frail participants (*p* < 0.05). Regarding comorbidities, the prevalence of hypertension (48.9%), diabetes (22.2%), and dyslipidemia (33.8%) was significantly elevated in frail participants than in non-frail participants, particularly within the 60–74-year age group (*p* < 0.05), although these associations showed varying significance in the oldest age group (≥75 years).

Body composition analysis revealed age-specific patterns: BMI was significantly higher in frail participants within the 25–44-year age group (25.22 ± 4.96 kg/m^2^) than in their non-frail counterparts (*p* = 0.013); however, this disparity diminished with age. Conversely, total body water and ASM differed significantly across age groups and according to oral frailty status (all *p* < 0.001). In frail participants, ASM decreased from 21.51 ± 5.35 kg (25–44 years) to 16.00 ± 3.84 kg (≥75 years). Notably, frail participants in the younger age groups (25–59 years) exhibited significantly higher ASM and water content than their non-frail peers did (*p* < 0.05), reflecting a different physical profile of oral frailty in the early stage compared with older ages (Table 2).

### 3.3. Impact of Oral Frailty on Mental Health and Quality of Life

Oral frailty was associated with higher psychological vulnerability and lower quality of life across the life course (Table 3). The prevalence of memory impairment in frail participants rose with age, peaking at 12.6% in the ≥75-year age group, with significant disparities between frail and non-frail participants in the 45–74-year age group (*p* < 0.05). Depression and perceived stress were significantly more prevalent in frail participants than in non-frail participants across all age groups (*p* < 0.05). Notably, perceived stress was the highest in frail participants in the 25–44-year age group (49.5%), suggesting a strong link between oral health and psychological burden in younger adults. Suicidal ideation was significantly more frequent among frail participants than in non-frail participants in the 45–59-year (8.0%) and ≥75-year (7.1%) age groups (*p* < 0.05). Health-related quality of life, measured by the HINT-8, was significantly lower (indicated by higher scores) in frail participants across all age groups (*p* < 0.001). Furthermore, generalized anxiety (assessed using GAD-7) was most severe in frail participants in the 25–44-year age group (3.67 ± 4.09), with significant differences observed between frail and non-frail participants in all age groups except the 60–74-year age group (*p* < 0.05; Table 3).

### 3.4. Correlations Between Oral Frailty and Health Indicators

The relationships between oral frailty-related factors and various health indicators were analyzed using Spearman’s rank correlation coefficients (Table 4). Health-related quality of life (assessed using HINT-8) demonstrated significant positive correlations with psychological distress, specifically depression (*r* = 0.425, *p* < 0.01) and generalized anxiety (assessed using GAD-7; *r* = 0.425, *p* < 0.01). Additionally, the HINT-8 score showed significant associations with physical health markers; it was positively correlated with activity limitations (*r* = 0.397, *p* < 0.01) and subjective poor oral health (*r* = 0.394, *p* < 0.01). In contrast, the HINT-8 score was negatively correlated with physiological markers, such as total body water content (*r* = −0.258, *p* < 0.01) and ASM (*r* = −0.264, *p* < 01). These findings indicate that greater oral frailty, characterized by lower muscle mass and poorer oral health, is closely linked to diminished mental health and overall quality of life across the life course (Table 4).

### 3.5. Relative Contribution of Oral Health Components to the Composite OFI

Multivariable linear regression analysis was performed to evaluate the relative contribution of individual oral health components to the composite OFI among Korean adults aged ≥25 years (Table 5). The model was statistically significant (F = 2620.700, *p* < 0.001). Among the oral health components, pronunciation discomfort (B = 2.310, *p* < 0.001), toothache experience (B = 1.027, *p* < 0.001), denture use (B = 0.975, *p* < 0.001), and subjective poor oral health (B = 0.514, *p* < 0.001) showed the largest relative contributions to the composite OFI. Speaking difficulty (B = 0.195, *p* < 0.001) made a significant contribution to the composite OFI. A greater number of natural teeth was inversely associated with the composite OFI (B = −0.052, *p* < 0.001). In addition, body composition index (B = 0.007, *p* = 0.006) and poorer health-related quality of life (assessed using HINT-8; B = 0.008, *p* = 0.003) were significantly associated with higher OFI (Table 5).

## 4. Discussion

In this study, we investigated the multidimensional nature of oral frailty and its association with physical, mental, and socioeconomic factors using a nationally representative sample of Korean adults. Our findings revealed that oral frailty is not merely a consequence of aging but a complex geriatric syndrome intertwined with systemic health and socioeconomic status [5,20].

### 4.1. Socioeconomic Disparities and Oral Frailty Across the Life Course

The prevalence of oral frailty increased significantly with age, reaching 48.5% in individuals aged ≥75 years. However, our results emphasize that socioeconomic vulnerability—specifically lower education and Medical Aid coverage—acts as a strong determinant of oral frailty, consistent with previous research on health inequities [24]. Notably, these disparities were evident even among younger adults (25–44 years), among whom Medical Aid recipients showed a substantially higher prevalence of oral frailty than NHI beneficiaries did. This finding suggests that the long-term accumulation of oral health disadvantage may begin well before old age [6]. Furthermore, it is consistent with previous evidence indicating that socioeconomic disadvantage and reduced access to preventive dental care contribute to oral health inequalities throughout adulthood [25]. Therefore, oral frailty may reflect not only age-related functional decline but also cumulative social disadvantage, highlighting the importance of life-course-oriented oral health promotion and targeted preventive interventions for socioeconomically vulnerable populations.

### 4.2. Interplay Between Physical Health, Sarcopenia, and Oral Function

A key finding of this study was the close association between oral frailty and physical health indicators, particularly muscle mass. The observed decline in ASM among frail participants is consistent with previous studies suggesting an interconnection between systemic muscle loss and impaired oral function, including the masticatory and suprahyoid muscles [15,22]. Furthermore, the higher prevalence of hypertension and diabetes among frail participants is consistent with previous evidence indicating a potential bidirectional relationship between chronic diseases and oral health [8]. Although causal relationships cannot be established in this cross-sectional study, chronic systemic conditions may co-occur with poorer oral health, and oral frailty may also be linked to nutritional deficiencies and unfavorable metabolic conditions [7,20]. In addition, the significant association between higher BMI and oral frailty observed among younger adults may reflect a metabolic profile of oral frailty that differs from the “thin-frail” pattern that is more commonly reported in older populations.

### 4.3. Psychological Vulnerability: The “Anxiety-Oral Frailty” Link in Young Adults

Distinctively, our study highlights the psychological burden associated with oral frailty across adulthood. Cognitive decline has been the primary focus of previous studies involving older adults [9]; however, in this study, younger participants (25–44 years) with oral frailty exhibited the highest levels of generalized anxiety (assessed using GAD-7). These findings suggest that oral functional limitations, particularly subjective speech or phonetic difficulties, may be closely linked to reduced social interaction and lower self-esteem among younger adults [10,11]. Although causality cannot be inferred in this cross-sectional study, oral frailty may represent an early correlate of psychological vulnerability and social withdrawal across the adult life course.

### 4.4. Relative Contribution of Oral Function Components: From Dentition to Function

The regression analysis indicated that speaking difficulty contributed more strongly to the overall OFI, compared with the number of natural teeth. These findings suggest that functional oral performance may be a more informative indicator of oral frailty, compared with structural measures, supporting a shift from a dentition-centered to a function-centered model [17]. This may be because speech production requires complex neuromuscular coordination, making impaired speech function a potentially sensitive indicator of early neuromuscular decline before overt physical frailty becomes clinically apparent [4,26].

### 4.5. Strengths and Limitations

The primary strength of this study is the use of a large-scale, nationally representative dataset (KNHANES), ensuring high generalizability to the Korean adult population. Furthermore, by including adults across a wide age range, in this study, we identified age-specific patterns of oral frailty throughout the adult life course, extending previous research that was primarily focused on older adults.

Despite these strengths, this study has limitations. First, because this study employed a cross-sectional design, causal relationships cannot be inferred, and the observed associations should be interpreted without implying temporal or causal direction. Future longitudinal studies are required to determine the temporal sequences and clarify whether oral frailty precedes sarcopenia or vice versa [21]. Second, although our operationalization of oral frailty utilized validated KNHANES items [13], compared with standardized clinical assessments, available survey variables may not fully capture all clinical dimensions of oral hypofunction (e.g., objective tongue pressure or masticatory performance). Thus, future research incorporating objective functional measurements is necessary to refine the global consensus on standardized diagnostic criteria [9]. Third, several oral health indicators were based on self-reported questionnaire data, which may have introduced recall or reporting bias. Lastly, although we adjusted for various socioeconomic and health-related confounders, residual confounding from unmeasured variables cannot be entirely excluded. Future longitudinal studies incorporating standardized clinical assessments and additional behavioral and clinical factors are warranted to validate the present findings, clarify the determinants and causal pathways of oral frailty, and determine whether early identification and intervention during young and middle adulthood can delay its progression across the adult life course.

## 5. Conclusions

Oral frailty was significantly associated with multiple physical and mental health indicators across adult age groups. Older age groups showed a higher prevalence of oral frailty than younger age groups. These findings provide evidence for understanding the multidimensional associations of oral frailty in Korean adults and may inform future longitudinal studies and age-specific oral health strategies.

## Figures and Tables

**Table 1:** Sociodemographic factors associated with oral frailty.

		25–44 Years (*n* = 1239)	45–59 Years (*n* = 1286)	60–74 Years (*n* = 1443)	≥75 Years (*n* = 491)	*p*
Oral frailty		101 (8.2)	213 (16.6)	405 (28.1)	238 (48.5)	0.000
Subjective oral health	Unhealthy	279 (22.5)	414 (32.2)	583 (40.4)	219 (44.6)	0.000
Denture use	Yes	1 (0.1)	26 (2.0)	189 (13.1)	185 (37.7)	0.000
Chewing discomfort	Yes	92 (7.4)	199 (15.5)	322 (22.3)	171 (34.8)	0.000
Toothache experience	Yes	345 (27.8)	395 (30.7)	410 (28.4)	112 (22.8)	0.000
Oral examination	Yes	669 (43.7)	696 (44.6)	795 (43.8)	176 (24.9)	0.000
Number of natural teeth		27.90 ± 2.11	26.21 ± 4.78	21.81 ± 7.73	15.93 ± 9.46	0.000 (all)
Chewing difficulty		1.78 ± 0.97	2.15 ± 1.11	2.40 ± 1.21	2.69 ± 1.34	0.000 (all)
Speaking difficulty		1.28 ± 0.58	1.48 ± 0.83	1.71 ± 1.02	2.00 ± 1.17	0.000 (all)
Toothbrushing frequency		2.10 ± 0.89 ^a^	2.20 ± 0.91 ^b^	2.30 ± 0.93 ^b^	2.14 ± 1.04 ^a^	0.000
Sex	Male	58 (10.5)	113 (21.4)	205 (32.5)	115 (49.8)	
	Female	43 (6.3)	100 (13.2)	200 (24.6)	123 (47.3)	
	*p*	0.009	0.000	0.001	0.589	
Education level	Middle school or less	3 (14.3)	30 (30.0)	245 (33.9)	194 (53.0)	
	High school graduate	34 (12.0)	105 (18.5)	117 (26.2)	38 (46.9)	
	College graduate or higher	64 (6.9)	78 (12.6)	43 (15.8)	6 (13.6)	
	*p*	0.013	0.000	0.000	0.000	
Type of insurance	National Health Insurance	96 (7.9)	200 (16.0)	358 (26.2)	205 (47.1)	
	Medical Aid	5 (26.3)	13 (39.4)	47 (62.7)	33 (58.9)	
	*p*	0.015	0.001	0.000	0.118	

Data are presented as *n* (%) or mean ± standard deviation (SD). Post hoc comparisons were performed using Bonferroni or Scheffé tests; different superscript letters indicate significant differences between groups (*p* < 0.05).

**Table 2:** Physical health indicators and oral frailty.

			25–44 Years	45–59 Years	60–74 Years	≥75 Years	Overall	*p*
Activity limitation	Non-frail		40 (3.5)	60 (5.6)	90 (8.7)	27 (10.7)	217 (6.2)	0.000
	Frail		10 (9.9)	22 (10.3)	76 (18.8)	64 (26.9)	172 (18.0)	0.000
	*p*		0.005	0.010	0.000	0.000		
Subjective poor health	Non-frail		153 (13.4)	162 (15.1)	175 (16.9)	41 (16.2)	531 (15.2)	0.161
	Frail		30 (29.7)	55 (25.8)	135 (33.3)	71 (29.8)	291 (30.4)	0.282
	*p*		0.000	0.000	0.000	0.000		
Chronic diseases	Hypertension	Non-frail	35 (3.1)	229 (21.3)	422 (40.7)	152 (60.1)	838 (23.9)	0.000
		Frail	6 (5.9)	50 (23.5)	195 (48.9)	142 (59.7)	396 (41.4)	0.000
		*p*	0.110	0.272	0.003	0.499		
	Diabetes mellitus	Non-frail	22 (1.9)	81 (7.5)	197 (19.0)	56 (22.1)	356 (10.2)	0.000
		Frail	1 (1.0)	33 (15.5)	90 (22.2)	54 (22.7)	178 (18.6)	0.000
		*p*	0.428	0.000	0.095	0.484		
	Dyslipidemia	Non-frail	48 (4.2)	243 (22.6)	439 (42.3)	108 (42.7)	838 (23.9)	0.000
		Frail	6 (5.9)	53 (24.9)	137 (33.8)	72 (30.3)	268 (28.0)	0.000
		*p*	0.273	0.266	0.002	0.003		
Body mass index (kg/m^2^)		Non-frail	23.93 ± 4.20 ^a^	24.13 ± 3.61	24.07 ± 3.11	23.78 ± 3.08	24.02 ± 3.64 ^d^	0.413
		Frail	25.22 ± 4.96	24.46 ± 3.60	24.09 ± 3.37	23.90 ± 3.39	24.25 ± 3.64	0.012
		*p*	0.013	0.217	0.907	0.692		
Total body water content		Non-frail	35.38 ± 8.06	33.45 ± 6.97	31.91 ± 6.06	30.13 ± 5.37	33.38 ± 7.19	0.000 (all)
		Frail	37.43 ± 8.65	34.75 ± 6.80	32.12 ± 5.94	29.75 ± 5.36	32.68 ± 6.76	0.000 (all)
		*p*	0.015	0.013	0.560	0.467		
Appendicular skeletal muscle mass		Non-frail	20.17 ± 5.12	18.80 ± 4.54	17.62 ± 4.12	16.31 ± 3.89	18.72 ± 4.73	0.000 (all)
		Frail	21.51 ± 5.35	19.62 ± 4.41	17.68 ± 4.03	16.00 ± 3.84	18.10 ± 4.55	0.000 (all)
		*p*	0.013	0.016	0.813	0.380		

Data are presented as *n* (%) or mean ± standard deviation (SD). Post hoc comparisons were performed using Scheffé tests; different superscript letters indicate significant differences between groups (*p* < 0.05).

**Table 3:** Impact of oral frailty on mental health and quality of life.

		25–44 Years	45–59 Years	60–74 Years	≥75 Years	Overall	*p*
Memory impairment	Non-frail	35 (3.1)	20 (1.9)	52 (5.0)	24 (9.5)	131 (3.7)	0.000 (all)
	Frail	5 (5.0)	10 (4.7)	32 (7.9)	30 (12.6)	77 (8.0)	0.011 (all)
	*p*	0.222	0.018	0.026	0.169		
Depression	Non-frail	87 (7.6)	46 (4.3)	30 (2.9)	5 (2.0)	168 (4.8)	0.000 (all)
	Frail	14 (13.9)	18 (8.5)	31 (7.7)	26 (10.9)	89 (9.3)	0.194
	*p*	0.029	0.012	0.000	0.000		
Suicidal ideation	Non-frail	53 (4.7)	36 (3.4)	36 (3.5)	8 (3.2)	133 (3.8)	0.325
	Frail	8 (7.9)	17 (8.0)	20 (4.9)	17 (7.1)	62 (6.5)	0.407
	*p*	0.116	0.003	0.127	0.035		
Perceived stress awareness	Non-frail	383 (33.7)	243 (22.6)	148 (14.3)	29 (11.5)	803 (22.9)	0.000 (all)
	Frail	50 (49.5)	76 (35.7)	82 (20.2)	46 (19.3)	254 (26.5)	0.000 (all)
	*p*	0.001	0.000	0.004	0.017		
Sleep duration	Non-frail	6.73 ± 1.16 ^a^	6.63 ± 1.15 ^ac^	6.57 ± 1.25 ^c^	6.58 ± 1.36 ^ac^	6.64 ± 1.20	0.012
	Frail	6.57 ± 1.15	6.47 ± 1.33	6.37 ± 1.45	7.52 ± 10.48	6.70 ± 5.37	0.060
	*p*	0.187	0.161	0.014	0.172		
Activity limitation & quality of life (assessed using HINT-8)	Non-frail	12.81 ± 3.11 ^a^	13.08 ± 3.22 ^a^	13.89 ± 3.53 ^b^	14.58 ± 3.87 ^b^	13.34 ± 3.38	0.000
	Frail	14.69 ± 3.85 ^a^	14.38 ± 3.73 ^a^	15.33 ± 4.12 ^b^	16.86 ± 4.73 ^c^	15.43± 4.26	0.000
	*p*	0.000	0.000	0.000	0.000		
Generalized Anxiety Disorder (assessed using GAD-7)	Non-frail	2.52 ± 3.33 ^a^	1.92 ± 3.17 ^b^	1.37 ± 2.92 ^c^	0.92 ± 2.11 ^c^	1.88 ± 3.13	0.000
	Frail	3.67 ± 4.09 ^a^	2.79 ± 4.48 ^a^	1.58 ± 2.98 ^b^	2.11 ± 4.12 ^b^	2.20 ± 3.82	0.000
	*p*	0.007	0.007	0.227	0.000		

Data are presented as *n* (%) or mean ± standard deviation (SD). Post hoc comparisons were performed using Scheffé tests; different superscript letters indicate significant differences between groups (*p* < 0.05). HINT-8, Health-related Quality of Life Instrument with 8 Items; GAD-7, Generalized Anxiety Disorder-7.

**Table 4:** Correlation between oral frailty and health/mental health indicators.

	1	2	3	4	5	6	7	8	9	10	11	12	13	14	15
1. Health-related quality of life (assessed using HINT-8)	1.000														
2. Activity limitation	0.397 **	1.000													
3. Subjective oral health	0.394 **	0.236 **	1.000												
4. Hypertension	0.136 **	0.065 *	0.076 *	1.000											
5. Diabetes mellitus	0.062	0.035	0.035	0.236 **	1.000										
6. Hyperlipidemia	0.132 **	0.054	0.117 **	0.355 **	0.288 **	1.000									
7. Body mass index	−0.016	−0.019	0.010	0.153 **	0.091 **	0.132 **	1.000								
8. Total body water content	−0.258 **	−0.109 **	−0.054	−0.048	0.062	−0.060	0.355 **	1.000							
9. Appendicular lean mass	−0.264 **	−0.117 **	−0.051	−0.076 *	0.037	−0.075 *	0.282 **	0.987 **	1.000						
10. Memory impairment	0.358 **	0.202 **	0.048	0.024	0.046	0.029	−0.013	−0.114 **	−0.120 **	1.000					
11. Depression	0.425 **	0.272 **	0.183 **	0.009	−0.024	0.041	−0.007	−0.099 **	−0.096 **	0.249 **	1.000				
12. Suicidal ideation	0.243 **	0.164 **	0.128 **	0.003	0.060	0.025	0.006	−0.039	−0.043	0.203 **	0.383 **	1.000			
13. Perceived stress awareness	0.319 **	0.092 **	0.179 **	−0.067 *	−0.053	0.036	0.041	0.061	0.070 *	0.156 **	0.321 **	0.270 **	1.000		
14. Average weekday sleep duration	−0.187 **	−0.077 *	−0.147 **	−0.028	0.035	−0.047	−0.029	0.102 **	0.103 **	−0.025	−0.092 **	−0.085 **	−0.184 **	1.000	
15. Generalized anxiety disorder (assessed using GAD-7)	0.425 **	0.204 **	0.204 **	−0.075 *	0.005	0.042	−0.002	−0.007	0.005	0.149 **	0.391 **	0.283 **	0.470 **	−0.116 **	1.000

Correlation coefficients were calculated using Spearman’s rank correlation analysis. * *p* < 0.05; ** *p* < 0.01 (two-tailed). HINT-8, Health-related Quality of Life Instrument with 8 Items; GAD-7, Generalized Anxiety Disorder-7.

**Table 5:** Relative contribution of oral function components to the composite OFI.

	B	SE	β	t	*p*	95%	
						LLCI	ULCI
Age	−0.004	0.001	−0.030	−4.471	<0.001	−0.005	−0.002
Sex (female)	−0.159	0.019	−0.043	−8.533	<0.001	−0.195	−0.122
Education level	−0.068	0.010	−0.045	−7.089	<0.001	−0.087	−0.049
Type of health insurance	−0.013	0.005	−0.014	−2.744	0.006	−0.022	−0.004
Number of teeth	−0.052	0.002	−0.203	−27.095	<0.001	−0.056	−0.049
Denture use	0.975	0.043	0.151	22.570	<0.001	0.890	1.060
Chewing difficulty	−0.062	0.014	−0.040	−4.421	<0.001	−0.089	−0.034
Pronunciation discomfort	2.310	0.038	0.476	60.421	<0.001	2.235	2.385
Speaking difficulty	0.195	0.013	0.096	15.397	<0.001	0.170	0.219
Toothache experience	1.027	0.021	0.250	49.056	<0.001	0.986	1.068
Tooth brushing frequency	−0.003	0.001	−0.012	−2.418	0.016	−0.006	−0.001
Subjective poor oral health	0.514	0.013	0.214	39.280	<0.001	0.489	0.540
Body composition index	0.007	0.002	0.013	2.741	0.006	0.002	0.012
Health-related quality of life (assessed using HINT-8)	0.008	0.003	0.016	2.984	0.003	0.003	0.013

F = 2620.700 (*p* = 0.000), R^2^ = 0.898, _adj_R^2^ = 0.898, D-W = 1.926. OFI, Oral Frailty Index; LLCI, lower limit of confidence interval; ULCI, upper limit of confidence interval; HINT-8, Health-related Quality of Life Instrument with 8 Items. Reference categories: male (sex); ≤ middle school (education level); Medical Aid (type of health insurance); and no denture use (denture use). Statistical analysis: multiple linear regression.

## Data Availability

Publicly available datasets were analyzed in this study. These data can be found at the Korea National Health and Nutrition Examination Survey (KNHANES) website provided by the Korea Disease Control and Prevention Agency.

## Abbreviations

The following abbreviations are used in this manuscript:

ASM	Appendicular skeletal muscle mass
BMI	Body mass index
CI	Confidence interval
GAD-7	Generalized Anxiety Disorder-7
HINT-8	Health-related Quality of Life Instrument with 8 Items
KDCA	Korea Disease Control and Prevention Agency
KNHANES	Korea National Health and Nutrition Examination Survey
NHI	National Health Insurance
OFI	Oral Frailty Index
